# Dr. E. L. Childs

**Published:** 1896-12

**Authors:** G. Alden Mills

**Affiliations:** New York


### DR. E. L. CHILDS.

Dr. E. L. Childs died at Conway, Mass., May 27, 1876, of cancer of the mesentery.

He was a native of Massachusetts and studied dentistry with Dr. E. Lincoln Clark, now of Dubuque, Iowa. He commenced practice in Pittsfield, Mass., and subsequently removed to Brooklyn, N. Y., and again to Nebraska, when failing health compelled a return to his former home in New England among the mountains of Conway.

My first acquaintance with Dr. Childs was in the city of Brooklyn. When the Brooklyn Dental Society was organized he engaged in its work with much enthusiasm. This society has never been excelled for its usefulness in advancing real professional merit, and the profession in Brooklyn owe much to it for its advanced standing in dentistry.

To Dr. Childs's work we accord a large share of approbation for the part he was always ready to perform. This society being one of our own creation, we are pleased to pay a just tribute to those who were associated with us in its successful development.

G. Alden Mills.

New York.

